# Effect of Sample Sources on Heavy Metal Concentration Measured in Beta Vulgaris Organs

**DOI:** 10.1155/2022/4968739

**Published:** 2022-06-11

**Authors:** Oscar E. Rodríguez, Diana R. Hernández, William A. Andrade, Crispín A. Celis, Luis M. Pombo, Aníbal A. Teherán, Sandra P. Forero, Javier R. Velandia, Fabio E. Díaz

**Affiliations:** ^1^Environmental Engineering Program, Engineering School, Universidad El Bosque, CHOC-IZONE Research Group, Bogotá, Colombia; ^2^Chemistry Department, Sciences School, Pontificia Universidad Javeriana, GIFUJ Research Group, Bogotá, Colombia; ^3^GIFVTA and COMPLEXUS Research Groups, Fundación Universitaria Juan N. Corpas, Bogotá, Colombia; ^4^Environmental Management Group, Engineering School, Universidad EAN, Bogotá, Colombia; ^5^Civil Engineering School, Universidad Santo Tomás, GIFIC Research Group, Bogotá, Colombia

## Abstract

**Aim:**

Heavy metal concentration [mg/dL, MP] in soil and the transfer to vegetable organs may have a sampling effect. We compared the [MP] in soil and organ samples of *Beta vulgaris* collected in sites with socioeconomic differences potentially inducing phytotoxicity.

**Materials and Methods:**

Samples of Beta vulgaris and soils (*n* = 4 per sample of soil and plant material) were randomly collected from two distant geographic areas (Mosquera and Sibaté, Cundinamarca, Colombia). We determined the [MP] using acid digestion of HCl : HNO_3_ [1 : 1]; the [MP] was obtained by atomic absorption in Varian AA-140 and Shimadzu AA-7000 equipment. A two-way ANOVA estimated the effect (partial *η*2) of the sampling site and metal type on the [MP] and transfer to the vegetable.

**Results:**

In Sibaté, the means (SD) of As_1.44 (0.18), Co_1.09 (0.51), Cr_6.21 (0.33), Ni_0.22 (0.02), and Pb_4.17 (0.87) were higher than in Mosquera (As_1.06 (0.21), Co_0.81 (0.19), Cr_3.72 (0.51), Ni_0.13 (0.04), and Pb_1.69 (0.40)) (*p* value <0.05). The effect of the interaction between the metal type and *Beta vulgaris* organs on the [MP] (0.801) in Sibaté was more meaningful than in Mosquera (0.430). Additionally, there was a strong correlation (Spearman's *ρ* > 0.8, *p* value <0.001) between [MP_soil] and [MP_plants] and between the transfer of metals to the plant and to the leaves. *Discussion*. The sampling location has a differential effect on the [MP] in soil and the transfer to *Beta vulgaris*. Given the differential effect described, the monitoring and phytoremediation strategies must be adjusted to scenarios with potentially phytotoxic conditions.

## 1. Introduction

The quality of commonly consumed agricultural commodities depends directly on water sanitation, air and atmospheric variables, the raw material used for inducing and maintaining crops, and soil quality [1–3]. Soils with metal concentrations higher than the upper permissible limits (UPL) decrease organic matter and filtering capacity and produce adverse effects on biomass turnover, the reproduction rate of leaves, and plant mass [4, 5].

Metals such as zinc (Zn), copper (Cu), nickel (Ni), and magnesium (Mg) are part of the organo-mineral matrix of soils and play essential roles as macronutrients or micronutrients [6]. However, UTMS can be toxic; for instance, a phytotoxic effect was noted in lettuce, manifested as a decrease in the length of shoots and roots [7]. In green cabbage seed germination bioassays, secondary phytotoxicity was demonstrated at high Zn, Cu, and Ni concentrations in the soil [8].

UPL have been identified and occasionally linked to social factors such as industrialization in rural areas producing fruits or vegetables close to water basins, a potentially phytotoxic social setting [1, 2, 9]. Additionally, wastes from pharmaceutical products, pesticides, herbicides, organic fertilizers, and soil amendments are common vectors that, associated with soluble vehicles mobilized in river or wastewater, increase metal concentration in agricultural soils [5, 8, 10].

The problem of UPL has been described in countries with different economic income levels, highlighting determining factors such as the use of wastewater for irrigating crops, mainly vegetables [1, 9–12]; air pollution, transformed into atmospheric deposition with high nickel (Ni), lead (Pb), and cadmium (Cd) concentrations [11, 12]; the proximity of crops to mining areas or industrial complexes, related to high copper (Cu) and arsenic (As) concentrations [7, 11, 12]; and the use of livestock manure as a crop fertilizer, associated with high mercury concentrations [12].

Besides the association with UPL, the determinants of the transfer of metals from soil or air to roots, stems, leaves, and fruits comprise a process that ends up in phytotoxicity [9, 10]. The transfer from the soil directly affects Cd, Cu, Pb, Ni, Cr, Mn, and Zn concentration in fruits, leaves, and roots of different vegetables [9, 13, 14].

Geomorphological aspects and potentially phytotoxic social settings may be determining factors in the transfer of metals to plant organs. In Colombia, we compared the effect of different vegetables on the percentage of metal transfer; nonetheless, it is necessary to study the effect of sampling sites [9]. This research compares metal concentration in the soil, different organs of a vegetable, and the transfer to the vegetable (overall) and the leaves on two sampling sites with similar environmental characteristics but socioeconomically different, increasing the probability of phytotoxicity.

## 2. Materials and Methods

### 2.1. Sampling Areas

**Figure 1:**
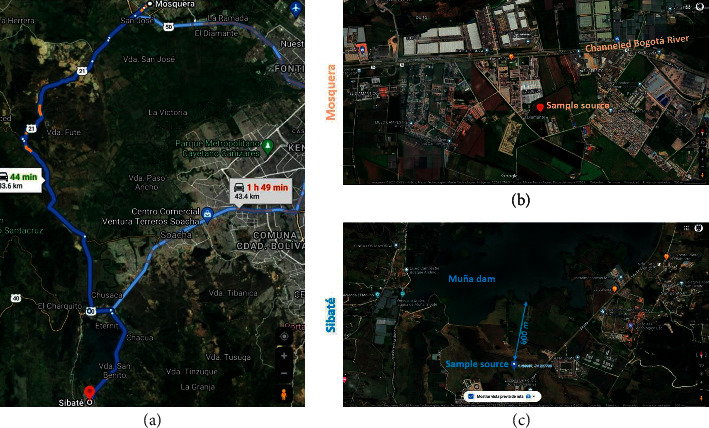
The map (a) shows the distance (by road) between Mosquera and Sibaté. (b, c) The maps of the Mosquera and Sibaté municipalities with nearby water basins.

Random samples were collected from *Beta vulgaris* leaves, stems, roots, and soil surrounding the vegetable in two geographic areas separated by 33.1 km (Mosquera, Sibaté_Vereda La Unión), located in the Cundinamarca department, Colombia. The annual average temperature in Mosquera (4°41′39.3″N, 74°11′32.1″W; 2,516 MASL) and Sibaté (4°30′03″N, 74°15′52″W; 2,570 MASL) ranges between 7–20°C and 6–18°C, respectively; both municipalities have 8-9 months of rain per year and economies strengthened by the agro-industrial and livestock sectors. In Mosquera, the cultivation of vegetables is the primary agricultural activity, and the Balsillas River is the primary water source (Map of collection sites). Meanwhile, in Sibaté, the crops of strawberries, potatoes, and vegetables are the primary agricultural products, and the Muña and Aguas Claras rivers are the primary water sources; however, contrary to Mosquera, the metallurgical, textile, and rubber industry has grown in the last two decades and become essential economic resources (Map of collection sites) (Figure 1).

### 2.2. Sampling

Samples of *Beta vulgaris* leaves, stems, roots, and soil surrounding the vegetable were obtained at random. We collected four plants per sampling point for the study; and four sampling points forming a square were considered, thus ensuring coverage of the entire crop area.

### 2.3. Sample Preparation

For determining heavy metals, acid digestion was carried out taking 10 g of fresh plant material (leaves, stems, and roots) and soil with 10 ml of a 1 : 1 mixture of HCl : HNO_3_ by refluxing for 24 hours; subsequently, the solutions were brought to a final volume of 50 ml with type 1 water.

### 2.4. Determination of Heavy Metals

Cu, As, Pb, Cr, Zn, Co, Cd, and Ni concentrations were determined by atomic absorption in Varian AA-140 and Shimadzu AA-7000 equipment, operated in the flame mode with air and acetylene. We established the gas flow and burner height conditions as recommended in the operation manuals and the instrument's default programs; each metal's optimal detection ranges are shown in the Table S2. The calibration curves for each metal were achieved with MERCK standards (1,000 ppm).

### 2.5. Statistical Analysis

Data were analyzed independently by sampling location. Due to the nature of the variables, metal concentration in plant organs or the soil was expressed in means (SD; SEM) and the percentage of transfer from the soil to the plant or leaves in medians (25th–75th). The mean concentration of each metal was compared with the maximum limit internationally established for vegetables with a *t*-test (right-tailed) [15]. The mean metal concentrations in the *Beta vulgaris* organs or the soil were compared between sampling sites with the *t*-test (one-tailed); the medians of metal transfers from the soil to the plant or leaves at the sampling sites were compared with the Mann–Whitney *U* test. Besides, we determined the correlation between metal concentration in the soil and leaves (Spearman's *ρ* (rho)). A *p*value <0.05 was established as significant.

An ANCOVA (Post hoc Bonferroni's test) test was performed to determine the interaction between various metal types and the *Beta vulgaris* organs at each sampling site and the effect on metal concentration. Another ANCOVA was run to determine the interaction between the different metal types and the sampling site and the effect on metal concentration. Before running the ANCOVAs, the metal concentrations determined in the plant organs and the soil were normalized independently for each sampling site using the Johnson transformation.

## 3. Results

### 3.1. General Characteristics

Two hundred twenty-four tests were performed to determine the concentration [mg/dL] of seven metal types in samples obtained from the *Beta vulgaris* root, aerial parts, and samples collected from the surrounding soil of the root.

**Figure 2:**
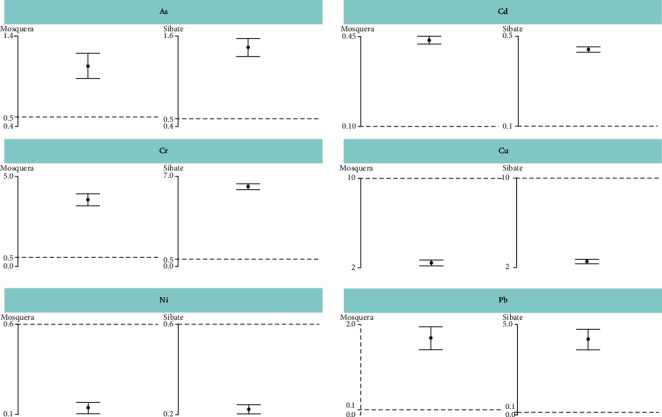
Comparison of the determined metal concentration with reference limits. The maximum metal concentrations in vegetables were obtained from CODEX (15); cadmium (Cd): 0.2 mg/kg, arsenic (As): 0.5 mg/kg, lead (Pb): 0.1 mg/kg, nickel (Ni): 0.6 mg/kg, copper (Cu): 10 mg/kg, and chrome (Cr): 0.5 mg/kg.

Table 1 describes the mean metal concentrations (SD) overall, by collection site, plant organ, and surrounding soil. In addition, in Figure 2, we compare the overall metal concentration determined at each collection site, with guideline values (reference limits); the mean As, Cd, Cr, and Pb concentrations were higher than the UPL (*p*value <0.01); the mean Cu and Ni concentrations were lower than the UPL, and for Co and Zn, there was no internationally established reference (Table 1, Figure 2).

**Figure 3:**
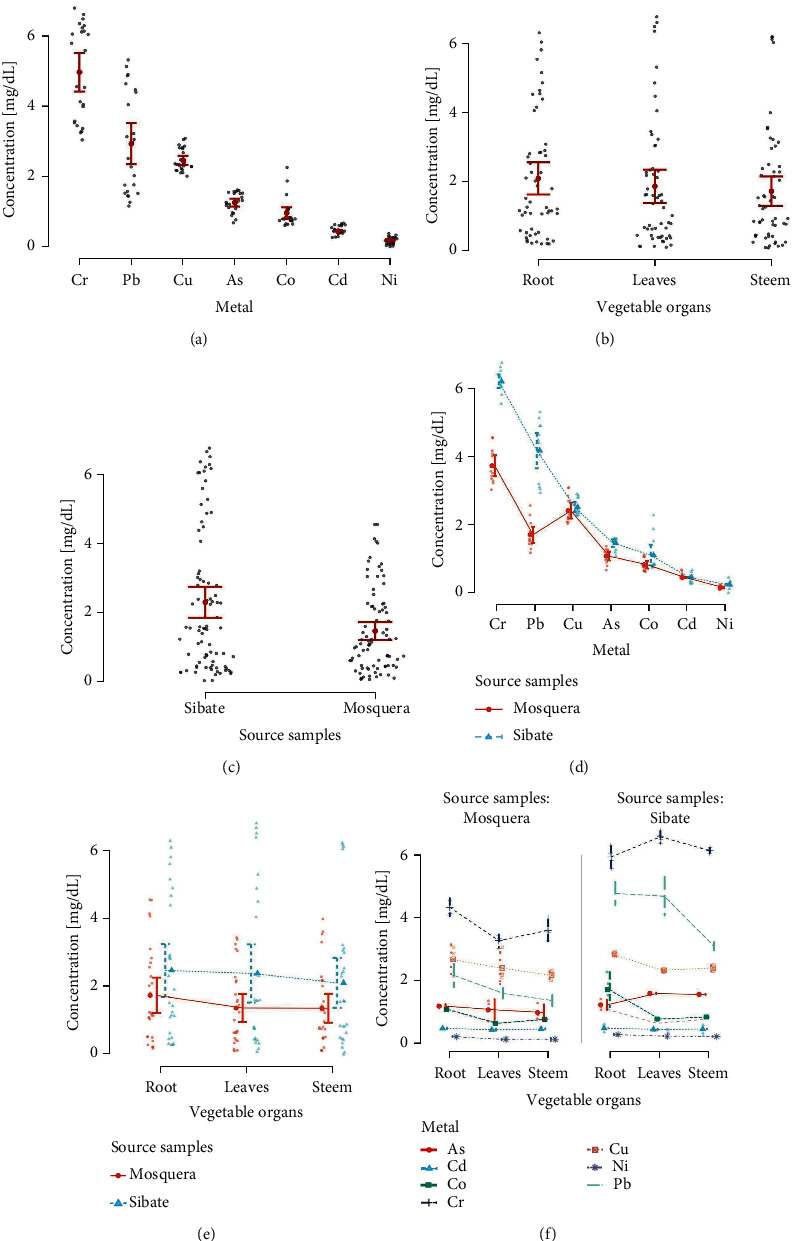
Heavy metal concentration overall, in parts of the plant, and by sampling place. Global distributions (a) of metal concentrations, (b) in the organs of *Beta vulgaris*, and (c) in the sampling sites. (d–f) The bivariate distributions of metal concentrations. The ranges of concentrations represent mean ± SEM. Global distributions (a) of metal concentrations, (b) in the organs of Beta vulgaris, and (c) in the sampling sites. (d–f) The bivariate distributions of metal concentrations. The ranges of concentrations represent mean ± SEM.

In addition, Figure 3(a) shows the distribution of the overall metal concentration and in the chard organs. In the distribution of measurements (mg/dL), we observe the minimum and maximum Cr (3.03–6.77), Pb (1.81–5.31), Cu (2.02–3.06), As (0.66–1.58), Co (0.60–2.26), Cd (0.39–0.47), and Ni (0.07–0.28) concentrations.

### 3.2. Metal Concentration in Plant Organs and Collection Sites

Table 1 compares “head-to-head” specific metal concentrations in the chard organs and the soil obtained from the sampled sites.

The concentrations were higher in Sibaté in general (Figure 3(c)) and for each metal, except Cd (Table 1, Figure 3(d)). When comparing the mean concentrations [SD] of the root (2.08 [1.78]), stem (1.71 [1.62]), and leaves (1.85 [1.84]) in “pool” (not classified by metal), we found no differences between the chard organs (one-way ANOVA, *Df*: 2; *F*: 0.646; *p* value: 0.525) (Figure 3(b)); nevertheless, Figure S1 shows asymmetric distributions of metal concentrations in the parts of the plant; for example, the highest concentrations of As were identified in the leaves, the highest Cu and Co concentrations in the root, and the highest Pb concentrations both in the leaves and the root.

In addition, the As, Co, Cr, Ni, and Pb concentrations in the leaves, stems, and roots were higher in Sibaté, except that the As concentration in roots did not show differences when comparing the sampled sites (Table 1, Figures 3(e) and 3(f)).

Finally, the soil samples showed higher Cd, Co, Cr, Ni, and Pb concentrations in Sibaté; Cu concentration was higher in Mosquera, and there were no differences in As concentration (Table 1).

### 3.3. Transfer of Metals from the Soil to the Plant

**Figure 4:**
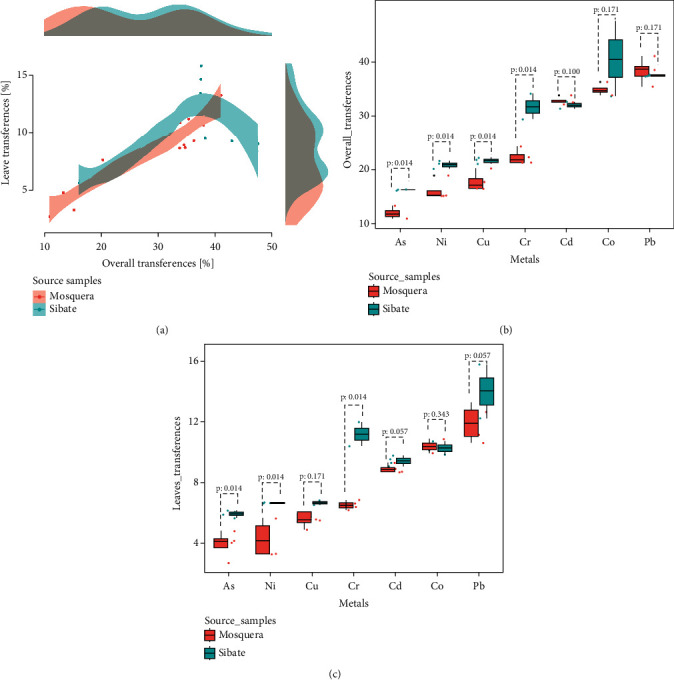
Transfer of metals to the plant and comparison between the sampling sites. Figure 4(a) presents a scatter plot between the transfer of metals to the plant and the leaves at both sampling sites. Figures 4(b) and 4(c) compare the transfer medians of each metal to the entire plant and the leaves in each sampling place; the comparisons were one-tailed, oriented towards the highest transfer median (Mann–Whitney *U*). In the three figures, navy blue represents the measurements calculated in Sibaté and pink the Mosquera measurements.

Table 2 and Figure 4 show the medians of transfers from the surrounding soil to the root of *Beta vulgaris* to the plant or leaves. In Mosquera, Pb was the metal with the highest median of transfer to the entire plant (*p*value <0.05, data not shown) and the leaves (*p*value <0.05, data not shown). In Sibaté, Co and Pb exhibited the highest medians of transfer to the plant (*p*value <0.05, Table S1), and Pb, the lowest median of transfer to the leaves (*p* value <0.05, Table S1). In both sampling sites, As was the metal with the lowest median transfer to the plant overall and the leaves (Table 2).

Generally, we determined a high correlation between metal concentration in the soil and the concentration in the plants (Spearman's *ρ*, 95%, *CI*: 0.946, 0.908–0.968; *p*value <0.001) and a high correlation between the transfer of metals to the plant and the transfer of metals to the leaves (Spearman's *ρ*, 95%, *CI*: 0.877, 0.798–0.926; *p*value <0.001) (Figure 4(a)).

In Mosquera and Sibaté, respectively, we found a high correlation between metal concentration in the soil and metal concentration in the plants (Spearman's *ρ*, 95%, *CI*: 0.95; *p*value <0.001). However, the correlation between the transfer of metals to the plant and the leaves was higher in Mosquera (Spearman's *ρ*, 95%, *CI*: 0.938, 0.868–0.971; *p* value <0.001) than in Sibaté (Spearman's *ρ*, 95%, *CI*: 0.799, 0.606–0.903; *p* value < 0.001).

Lastly, it was determined that the medians of transfers of As, Ni, Cu, and Cr to the entire plant and the leaves were higher in Sibaté. When comparing sampling sites, the median transfer of Co to the entire plant was also higher in Sibaté but without differences in the transfer to the leaves. No differences were identified in the general medians of Cd and Pb transfers between the sampling sites (Figure 4(b) and 4(c)). Of note is that, when estimating the difference in metal transfer medians between the two sampling sites, Cr was the metal with the most significant difference in the entire plant and the leaves (Table 2).

### 3.4. Interactions between Metals and Plant Organs

Given the differential distribution of concentrations and transfer of metals in the soil and the organs of *Beta vulgaris*, ANCOVA showed a significant effect on metal concentration secondary to the interaction between specific metals and the organs of the plant, highlighting that the effect was greater in Sibaté than in Mosquera (Table 3, Table S1). In addition, a significant effect was established on the metal concentration in the soil due to the interaction between specific metals and the sampling area (Table 3).

## 4. Discussion

This research compared metal concentration in the chard and the surrounding soil and the transfer to the plant organs in samples collected from two municipalities with similar environmental conditions but with potentially phytotoxicity-inducing socioeconomic differences.

Food security and its socio-environmental determinants, including soil pollution by poor agricultural practices, is a public health issue prioritized by the United Nations Food and Agriculture Organization (FAO). It promoted the World Soil Alliance to control, among others, overexploitation with synthetic chemicals and the use of wastewater as a primary water resource in irrigation to increase crop productivity [16, 17].

These control strategies are actions of the voluntary guidelines for sustainable soil management (SSM), which low-income or middle-income countries, including Colombia, can adopt and adapt to maintain healthy soils and contribute to food security [16]. In Colombia, the most recent legislation on the reuse of wastewater authorizes use after treatment processes in nonfood crops for humans or animals or food crops not intended for human or animal consumption subjected to physical or chemical processes. Nonetheless, reuse for irrigation activities in fruit and vegetable crops has been frequently documented [9, 18].

As described in other countries, studies conducted in Colombia are consistent in demonstrating high heavy metal concentrations in the organs of different types of vegetables and the surrounding soil of the root; however, the effect of the type of soil or samples collected on different geographic locations controlled in an experimental design has been studied little [3, 9, 19–21].

Our results prove an effect of the collection site on metal concentration in different parts of the plant and the soil, precisely the samples collected from Sibaté, a geographical setting with a triad of factors (agricultural activity, industrial activity, and proximity to water basins) that together induce phytotoxicity [21]. Nevertheless, the potential phytotoxic effect of this triad of factors requires a setting with environmental regulation problems, particularly a lack of monitoring and surveillance systems for wastewater reuse or the implementation of policies aimed at SSM [16, 22].

The impact of the activities and actions included in SSM has been described in research that determined the effect of good wastewater reuse practices (aerobic-anoxic treatment) or the application of soil amendments on metal concentration in the soil, vegetables, and fruits [22–24] Markers such as pH, cation exchange capacity, and the concentration of carbon and organic matter were higher in soils irrigated with treated water; conversely, the concentration of Fe, Mn, Zn, Pb, Cu, and Cd was higher in soils irrigated with treated water [23]. In our results, both geographic locations exhibited concentrations higher than the maximum permissible limits of As, Cd, Cr, and Pb in vegetables; however, in Sibaté, the interaction between the metal type and the organs of the vegetables showed an effect on metal concentration that doubled the estimate for Mosquera.

The effect on the metal concentration estimated in Sibaté can be explained by variables not measured in this research, including insecticides, herbicides, and nonorganic fertilizers in agricultural processes, or the types of industrial processes employed by the economic activity of each municipality. It may also be due to the differential implementation of healthy and standardized practices for the disposal of industrial waste or factors related to the flow or accumulation of metals in water basins, for example, the Muña reservoir in Sibaté, which is the primary water source for irrigating fruit and vegetable crops [25].

The location of the crop concerning reservoir-type water sources, which accumulate industrial waste in circumscribed geographical locations, can be compared to what happened in the province of Hunan, China. The collapse of a water dam immersed in an area of exploitation of Pb and Zn, and the subsequent discharge along a river that was a primary source for the irrigation of cereal and vegetable crops resulted in soils highly contaminated with Cd, Zn, Pb, and Cu after 17 years [25, 26]. As described, the continuous discharge for more than two decades and the reuse of untreated water for irrigating vegetable crops in Sibaté and other similar places in Colombia may have a long-term effect on the supranormal metal concentration in the soil and subsequent transfer to plants, even after implementing soil treatments that reverse or prevent these harmful effects [16, 18, 22, 25, 26].

A high correlation between the metal concentration in the soil and the vegetable was demonstrated; besides, Pb, Co, and Cd, in descending order, were the metals with the highest transfer rate to the vegetable overall and the leaves (Table 2, Figure 4). The metals with the highest transfer to the plants and leaves were different in each sampling site; in Mosquera, it was Pb, and in Sibaté, it was Co, which can be explained by the soil conditions in the sampling sites. For instance, in samples of soil obtained from farms using untreated wastewater to irrigate crops, it was shown that pH, the soil pollution index, and the interaction of some metals affect the transfer of Pb, Cr, Zn, Cu, C, and Ni to beets (*Beta vulgaris* L) [27].

In addition to the physicochemical characteristics or factors mentioned, factors not measured in this study, such as Fe concentration, chemical weathering of soils, and direct irrigation on the leaves of the plant, which have been strongly correlated with the concentration of Zn, Cu, Cr, and Ni in soil samples collected in riverine surroundings, may affect the transfer of metals from the soil to the entire vegetable or the leaves [28, 29].

With the results obtained, we can conclude that the sampling place has a differential effect on the concentration of different metal types in the soil and the subsequent transfer to the entire vegetable or the leaves. The strong correlation between the metal concentration in the soil and the plant and the correlations between the transfers of metals to the entire plant and the leaves can improve the efficiency of processes for monitoring and surveilling phytotoxicity by heavy metals secondary to the use of untreated wastewater as a primary irrigation resource in fruit and vegetable crops.

## Figures and Tables

**Table 1:** Mean metal concentrations (mg/kg).

Source samples	As	Cd	Co	Cr	Cu	Ni	Pb
Overall	1.25 (0.27)	0.43 (0.02)	0.95 (0.40)	4.97 (1.34)	2.45 (0.32)	0.18 (0.05)	2.93 (1.43)
M^†^	**1.06 (0.21)**	**0.43 (0.02)**	**0.81 (0.19)**	**3.72 (0.51)**	**2.40 (0.39)**	**0.13 (0.04)**	**1.69 (0.40)**
Leaves	1.05 (0.29)	0.41 (0.02)	0.62 (0.00)	3.26 (0.17)	2.39 (0.45)	0.10 (0.02)	1.58 (0.14)
Steam	0.96 (0.22)	0.44 (0.01)	0.75 (0.02)	3.59 (0.30)	2.16 (0.10)	0.11 (0.03)	1.35 (0.15)
Root	1.17 (0.05)	0.45 (0.01)	1.06 (0.02)	4.32 (0.25)	2.67 (0.41)	0.19 (0.01)	2.15 (0.32)
Soil	26.1 (2.57)	3.98 (0.12)	6.99 (0.13)	50.1 (1.35)	40.7 (1.64)^c^	2.55 (0.30)	13.2 (0.41)
S^†^	**1.44 (0.18)** ^**c**^	**0.44 (0.01)**	**1.09 (0.51)** ^a^	**6.21 (0.33)** ^**c**^	**2.51 (0.24)**	**0.22 (0.02)** ^**c**^	**4.17 (0.87)** ^**c**^
Leaves	1.57 (0.01)^b^	0.42 (0.01)	0.76 (0.02)^c^	6.57 (0.17)^c^	2.31 (0.04)	0.21 (0.00)^c^	4.68 (0.54)^c^
Steam	1.54 (0.00)^c^	0.43 (0.00)	0.82 (0.02)^b^	6.14 (0.06)^c^	2.38 (0.09)	0.19 (0.00)^b^	3.08 (0.11)^**c**^
Root	1.21 (0.14)	0.46 (0.00)	1.69 (0.49)^a^	5.94 (0.30)^c^	2.83 (0.05)	0.26 (0.01)^c^	4.76 (0.32)^c^
Soil	26.6 (1.12)	4.14 (0.02)^a^	8.06 (0.01)^**c**^	59.0 (2.06)^c^	34.7 (0.06)	3.20 (0.00)^b^	33.4 (0.21)^c^

^†^Municipalities, M: Mosquera, S: Sibaté. Means (SD). The letters ^abc^ indicate *p* values <0.05, <0.01, and <0.001, respectively. The mean metal concentrations of specific subgroups between Mosquera and Sibaté were compared; the letters were attached as a subscript in the subgroup where the mean concentration was higher (one-tail, independent samples *t*-test).

**Table 2:** Transfer of metals to plants and leaves.

Source samples	As	Cd	Co	Cr	Cu	Ni	Pb
Overall-M^†^	11 (11.0–13.0)	32 (32.2–33.4)	34 (33.9–35.8)	21 (21.3–23.7)	17 (16.4–19.6)	15 (15.1–17.9)	38 (36.0–40.4)
Overall-S^†^	16 (16.1–16.3)	31 (31.4–32.4)	40 (34.7–46.3)	31 (29.7–33.6)	21 (21.1–22.1)	20 (20.3–21.5)	37 (37.3–37.5)
∆M (95%CI)	4.5 (2.79–5.45)	NA	NA	9.5 (5.08–12.8)	4.5 (0.80–5.76)	5.4 (1.28–6.50)	NA
*To* leaves-M^†^	4.0 (3.03–4.61)	10 (9.99–10.7)	8.8 (8.68–9.20)	6.4 (6.22–6.77)	5.5 (5.02–7.10)	4.1 (3.26–5.47)	11 (10.7–13.0)
*To* leaves-S^†^	5.9 (5.69–6.12)	10 (9.90–10.6)	9.4 (9.10–9.70)	11 (10.5–11.8)	6.6 (6.54–6.77)	6.6 (6.61–6.66)	14 (12.5–15.4)
ΔM (95%CI)	1.8 (0.85–3.45)	NA	NA	4.6 (3.56–5.79)	NA	2.5 (0.97-3-40)	NA

^†^Municipalities, M: Mosquera, S: Sibaté. To leaves: metal transfer from the soil to leaves. Medians (25th–75th): medians in bold were compared to the medians of the rest of the relevant row. NA: no significant differences were identified.

**Table 3:** Effect of the interaction between heavy metals and plant organs.

Parameter	dF	*F*-statistic	*p* value	Partial *η*2
*Mosquera*				
Vegetable organs (Vo)	2	43.92	<0.001	0.582
Metal (M)	6	538.83	<0.001	0.981
Vo *∗* M	12	3.95	<0.001	**0.430**

*Sibaté*				
Vegetable organs (Vo)	2	41.82	<0.001	0.570
Metal (M)	6	1701.25	<0.001	0.994
Vo *∗* M	12	21.11	<0.001	**0.801**

*Soil*				
Source samples (SS)	1	23.02	<0.001	0.354
Metal (M)	6	157.24	<0.001	0.957
SS *∗* M	6	5.96	<0.001	**0.460**

Effect size—partial *η*2: Cohen's guidelines 0.2—small effect, 0.5—moderate effect, 0.8—large effect. Vo  ^*∗*^ M: vegetable organs  ^*∗*^ metal.

## Data Availability

The research data can be found at the following link: https://dataverse.harvard.edu/dataset.xhtml?persistentId=doi:10.7910/DVN/ODFDDJ.

## Supplementary Materials

Supplementary Materials Figure S1 shows asymmetric distributions of metal concentrations in the parts of the plant. The figure presents three columns of heavy metal concentration measurements made on leaves, roots, and stems (vegetable organs). Each measurement is presented as a point that identifies each metal with a specific color. Table S1 shows the post hoc statistical analysis between the plant organs and each metal. Table S2 shows optimal measurement ranges in linear range intervals obtained in Varian AA140 and Shimadzu AA7000 equipment.
