# Supplementary figures and images for: Effect of Sample Sources on Heavy Metal Concentration Measured in Beta Vulgaris Organs
Source: J Environ Public Health. 2022 Jun 11;2022:4968739. doi: 10.1155/2022/4968739 (PMC9206551; doi:10.1155/2022/4968739)

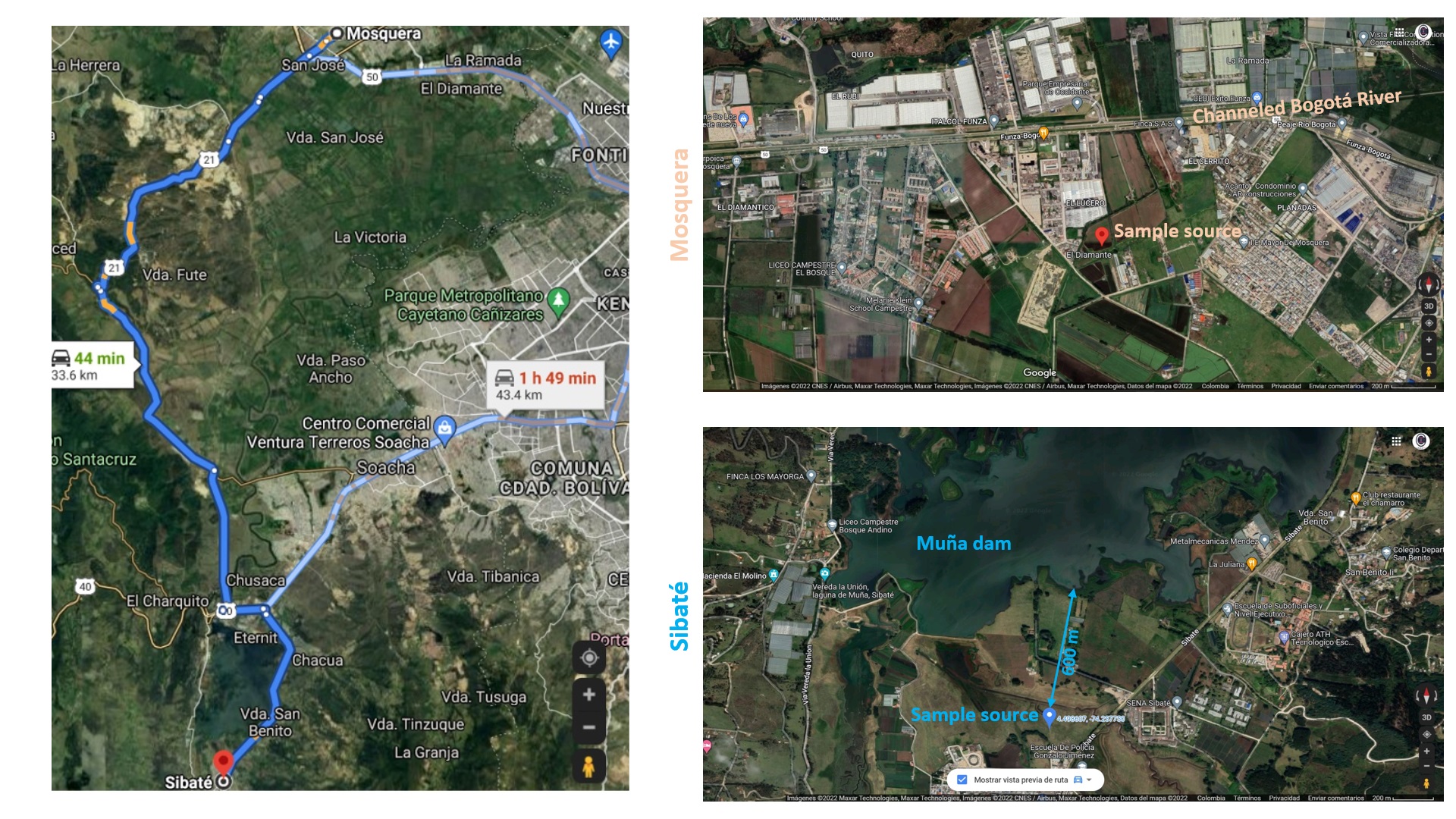

Supplement: Supplementary Materials — Figure S1 shows asymmetric distributions of metal concentrations in the parts of the plant. The figure presents three columns of heavy metal concentration measurements made on leaves, roots, and stems (vegetable organs). Each measurement is presented as a point that identifies each metal with a specific color. Table S1 shows the post hoc statistical analysis between the plant organs and each metal. Table S2 shows optimal measurement ranges in linear range intervals obtained in Varian AA140 and Shimadzu AA7000 equipment. [file 4968739.f1.zip › 4968739.f1/maps.jpg]

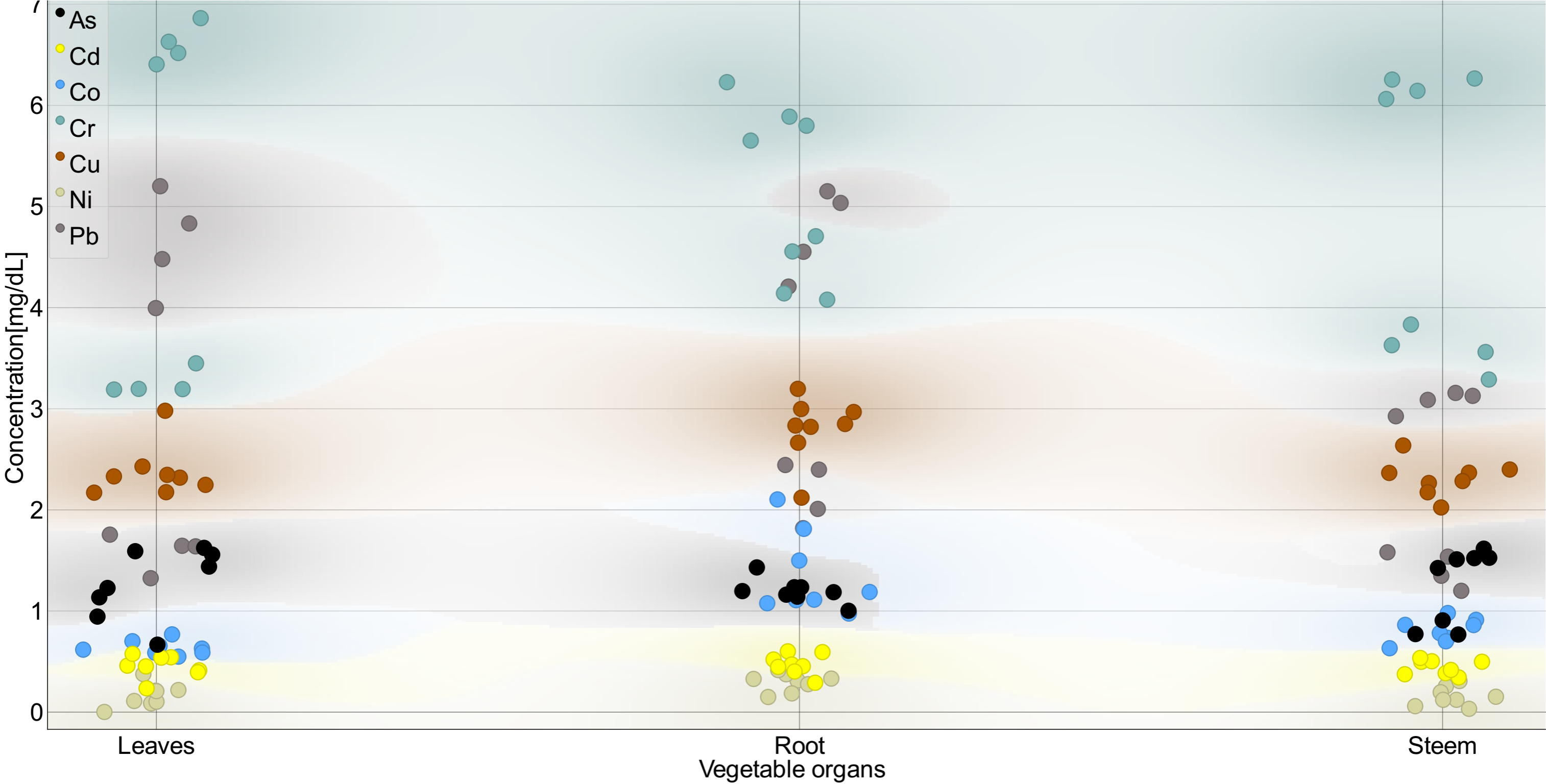

Supplement: Supplementary Materials — Figure S1 shows asymmetric distributions of metal concentrations in the parts of the plant. The figure presents three columns of heavy metal concentration measurements made on leaves, roots, and stems (vegetable organs). Each measurement is presented as a point that identifies each metal with a specific color. Table S1 shows the post hoc statistical analysis between the plant organs and each metal. Table S2 shows optimal measurement ranges in linear range intervals obtained in Varian AA140 and Shimadzu AA7000 equipment. [file 4968739.f1.zip › 4968739.f1/Supplementary.pdf]
